# The Effect of Corticosteroid Doses on Pain in Knee Osteoarthritis: A Systematic Review and Meta‐Analysis

**DOI:** 10.1002/msc.70121

**Published:** 2025-05-19

**Authors:** Marc‐Antoine Lafrenaye‐Dugas, Frédérique Dupuis, Valérie Bélanger, Marie‐Michèle Briand

**Affiliations:** ^1^ Department of Physical Medicine and Rehabilitation (Physiatry) Centre Hospitalier Universitaire de Québec – Université Laval Quebec City Canada; ^2^ Faculté de Médecine de l’Université de Sherbrooke Sherbrooke Canada; ^3^ Faculty of Medicine Université Laval Quebec City Canada; ^4^ Center for Interdisciplinary Research in Rehabilitation and Social Integration of Quebec Quebec City Canada; ^5^ Center of Research of the Hôpital du Sacré‐Coeur de Montreal CIUSSS du Nord‐de‐l’Île‐de‐Montreal Montreal Canada; ^6^ Faculty of Medicine Université de Montréal Montréal Canada

**Keywords:** corticosteroids, intra‐articular injection, knee, osteoarthritis (OA), pain

## Abstract

**Objective:**

To evaluate the effect of various doses of intra articular corticosteroid injection (IACI) on pain reduction in knee osteoarthritis when compared with normal saline and perform a between‐dose comparison.

**Methods:**

A systematic review with meta‐analysis was conducted searching four databases until April 2024. RCTs comparing the effect of IACI with normal saline on pain relief in knee osteoarthritis were included. The different doses were pooled into three categories: low (< 40 mg methylprednisolone equivalent), usual (40 mg), or high dose (> 40 mg). Their effect compared to normal saline was evaluated at very short (VST, 1–3 weeks), short (ST, 4–8 weeks) and middle term (MT, 10–16 weeks). A multivariate analysis carried out the influence of dosage on pain relief, at each time point. The Jadad scale was used to assess risks of bias and GRADE for certainty of evidence.

**Results:**

Eleven studies were included in the meta‐analyses (*n* = 1125 patients). Low dose was significantly superior to normal saline in the VST, but not in the ST (low‐quality evidence). No data were available for the MT. The usual dose was significantly superior to normal saline in the ST, but not in the VST and MT (moderate‐quality evidence). A high dose was significantly superior to normal saline in the ST and MT (low‐quality evidence). Multivariate analysis showed that the dose significantly influenced pain reduction at ST and MT, but not in the VST (low‐quality evidence).

**Conclusion:**

The dose of IACI doesn't influence pain reduction in the peak effect, but a higher dose seems to have a more prolonged effect.

**Abbreviations**
AAOS: American Academy of Orthopaedic Surgeons
ACR: American College of Rheumatology
BM: Betamethasone
BTA: Botulinum toxin type A
CIHR: Canadian Institute of Health Research
CZ: Cortivazol
FRQ‐S: Fonds de Recherche du Québec‐Santé
GRADE: Grading of Recommendations, Assessment, Development, and Evaluations
IACI: Intra‐articular corticosteroid injections
ICC: Intraclass correlation coefficient
INF: infliximab
KL: Kellgren–Lawrence radiological classification scale
MP: Methylprednisolone
MT: Middle term
OA: Osteoarthritis
PRP: platelet rich plasma
RCT: Randomized controlled trials
SD: Standard deviation
SMD: Standardized mean differences
ST: short term
TA: Triamcinolone acetonide
TH: Triamcinolone hexacetonide
VAS: Visual analog scale
VST: Very short‐term
WOMAC: Western Ontario and McMaster Universities Arthritis Index

## 1 Introduction

Osteoarthritis (OA) is the most common joint disorder with knees being the most affected joints (Allen et al. 2022). Intra‐articular corticosteroid injections (IACI) are frequently used by practitioners for pain management and their effectiveness has been demonstrated in previous reviews (Jüni et al. 2015; Najm et al. 2021; Saltychev et al. 2020). Hence, the latest guidelines of the American College of Rheumatology (ACR) (Kolasinski et al. 2020) and the American Academy of Orthopaedic Surgeons (AAOS) (Brophy and Fillingham 2022) have recommended the use of IACI for knee OA. However, IACI is associated with multiple adverse effects, such as flushing, hyperglycemia, hypertension, and hypothalamic‐pituitary‐adrenal axis suppression (Honcharuk and Monica 2016; Kamel et al. 2023; Stout et al. 2019), for which dosing seems to be an important risk factor (Johnston et al. 2015; Kamel et al. 2023).

To date, there is no consensus regarding the most optimal dosage for knee OA, and practitioners tend to use higher dosages for knee IACI (Lazaro et al. 2014). Few reviews (Cushman et al. 2018; Jüni et al. 2015) have attempted to evaluate the impact of dosing on the effectiveness of IACI in OA, but conclusions were limited by the limited evidence available. Considering the important possible dose‐dependant side effects of IACI and the lack of up‐to‐date reviews on the optimal dosage, it is relevant to evaluate the impact of dosing to support practice.

This systematic review with meta‐analysis aimed (1) to evaluate the effectiveness of different doses of intra‐articular corticosteroid injection (IACI) on pain reduction in knee osteoarthritis when compared to normal saline, and (2) to evaluate the influence of the dose on pain reduction. Adverse effects were also extracted as a secondary outcomes. The findings could help practitioners to determine the most effective dose that generates the best benefits while minimising adverse effects.

## 2 Methods

### 2.1 Search Strategy

Four databases were searched from their inception to April 2024: PubMed, Embase, Cochrane CENTRAL, and Web of Science. The search strategy suggested by Cochrane to identify randomized trials was used in PubMed and Embase (Higgins et al. 2019). The full search strategy is presented in Supporting Information S1: appendix 1. Relevant review papers identified during the search were subject to reference list checking and citation tracking to identify further studies meeting the inclusion criteria.

### 2.2 Study Eligibility

Articles presenting randomized controlled trials (RCT) with at least one IACI and one normal saline group of intra‐articular saline injections, and reporting outcomes related to pain intensity were included. Methylprednisolone or other corticosteroid with available equivalence (i.e., triamcinolone hexacetonide [TH]/acetonide [TA], betamethasone [BM]/dexamethasone sodium/acetate, or cortivazol [CZ] (Altman et al. 2019; Douglas 2012; Jüni et al. 2015; Michon et al. 2010; Stephens et al. 2008)) were included. Other corticosteroids (e.g., FX006) were excluded because there was no accepted equivalence. Accordingly, a dose less than 40 mg was considered low, and a dose greater than 40 mg was considered high. Every scale and tool for pain evaluation were accepted, but visual analog scale (VAS) was favoured for the analysis when present (Patel et al. 2021). Pain subscales of a functional tool were also considered (e.g., Knee injury and Osteoarthritis Outcome Score, Oxford knee score, Western Ontario and McMaster Universities Arthritis Index [WOMAC]). The total scores were considered only if no subscale score was available, with the condition of being highly correlated with the pain subscale (Bjerre‐Bastos et al. 2021; Papathanasiou et al. 2015).

Only studies with saline injections without local anaesthetic were included as controls group to allow comparison between studies. The study population was adults with a confirmed diagnosis of knee OA, regardless of the severity. French and English manuscripts were considered. Registered studies (e.g., clinicaltrial.gov) were considered eligible when complete methodology and results were available, if no article reporting the results of a relevant study was published yet.

Studies not reporting pain outcome after a single injection, involving joints other than the knee and OA from secondary causes (e.g., rheumatoid arthritis) were excluded.

### 2.3 Data Extraction

Data retrieved from databases were exported to Endnote (EndNote. EndNote 20 ed. Philadelphia, PA) for reference management. Covidence (Covidence systematic review software, Veritas Health Innovation, Melbourne, AUS) was used for duplicate removal and abstract screening. Two reviewers (M.L., F.D.) independently screened titles and abstracts and then full texts. Discrepancies between the reviewers were resolved by consensus. Data were extracted by one reviewer, using a data extraction form including information on the population, intervention, outcomes and adverse effects (see Supporting Information S1: appendix 2). When data were missing, the study's corresponding author was contacted by email.

### 2.4 Quality of Studies

The quality of the studies was assessed by two independent reviewers until consensus was reached, using the Jadad scale (Jadad et al. 1996). A third reviewer was consulted if no consensus was reached.

### 2.5 Quality of the Evidence

The GRADE (Grading of Recommendations, Assessment, Development, and Evaluations (Balshem et al. 2011)) approach was used by two independent reviewers to assess the quality of the evidence. The certainty of evidence was initially considered high (as RCTs were included) and rated down based on the risk of bias, imprecision, inconsistency, indirectness, and publication bias (Guyatt et al. 2011). Publication bias was assessed using funnel plots.

### 2.6 Data and Outcomes

To assess the effect on pain with a common effect size (ES) measure, mean changes from baseline comparing IACI groups to normal saline groups were converted into standardized mean difference (SMD) using the Wilson ES calculator (Wilson 2023). When standard deviation of change was not reported, it was estimated, considering the reliability of VAS and WOMAC (Alghadir et al. 2018; Basaran et al. 2010). When mean or standard deviation (SD) were missing in the manuscript and good quality graph was present, results were extracted from the graph using the pixels as a reference, a method that has been previously validated (see details in Supporting Information S1: appendix 3). (Van der Mierden et al. 2021).

Three‐time frames were chosen: very‐short term (VST: 1–3 weeks), short term (ST: 4–8 weeks) and middle term (MT 10–16 weeks), respectively corresponding to the peak effect (Jüni et al. 2015; Najm et al. 2021; Saltychev et al. 2020), the mean effect duration of IACI (i.e., triamcinolone and methylprednisolone) (Habib et al. 2010; Shah et al. 2019) and the usual waiting time before repeating an injection (Blankstein et al. 2021). Long‐term effect (> 3 months) were not evaluated, as usually not expected to be effective for IACI (Saltychev et al. 2020).

Three dosing categories were used based on guideline recommendations (Brophy and Fillingham 2022; Kolasinski et al. 2020). Usual dose was set at 40 mg of methylprednisolone (MP) dose‐equivalent (corresponding to 40 mg of triamcinolone hexacetonide (TH)/acetonide (TA), 8 mg of betamethasone (BM)/dexamethasone sodium/acetate, and 3 mg of cortivazol (CZ)) (Altman et al. 2019; Douglas 2012; Jüni et al. 2015; Michon et al. 2010; Stephens et al. 2008).

Adverse effects possibly or likely related to the injection as reported by the study's authors were extracted when available.

### 2.7 Statistical Analysis

First, a meta‐analysis with a‐effects model was used to evaluate the effect of the different doses on pain compared with normal saline. A mean SMD and 95% confidence intervals were obtained by pooling studies by dose categories (i.e., low, usual, and high dose) for every time frame (i.e., VST, ST, and MT). Results were presented as forest plots with a negative result representing pain reduction. Heterogeneity was assessed graphically with funnel plots and *I* ^2^ values for each time frame and dose category.

Second, a multivariate analysis with mixed effects was performed, with dose (i.e., low, usual and high) as a moderator to evaluate the influence of IACI dose on pain reduction (using the SMD previously obtained). This analysis was performed for each time frame (i.e., VST, ST and MT). Cohen's ES was used to detail the effects, with < −0.8 considered a high effect, −0.8 to −0.5 moderate, and ≥ 0.5 low (Lakens 2013).

Finally, a descriptive presentation of adverse effects was reported by dose categories as a percentage of the total number of participants.

All analyses were conducted using RStudio with Metafor project version 3.0‐2 (RStudio, PBC, Boston, MA, USA). Statistical significance was set at *p* < 0.05.

## 3 Results

### 3.1 Search Results

**FIGURE 1:**
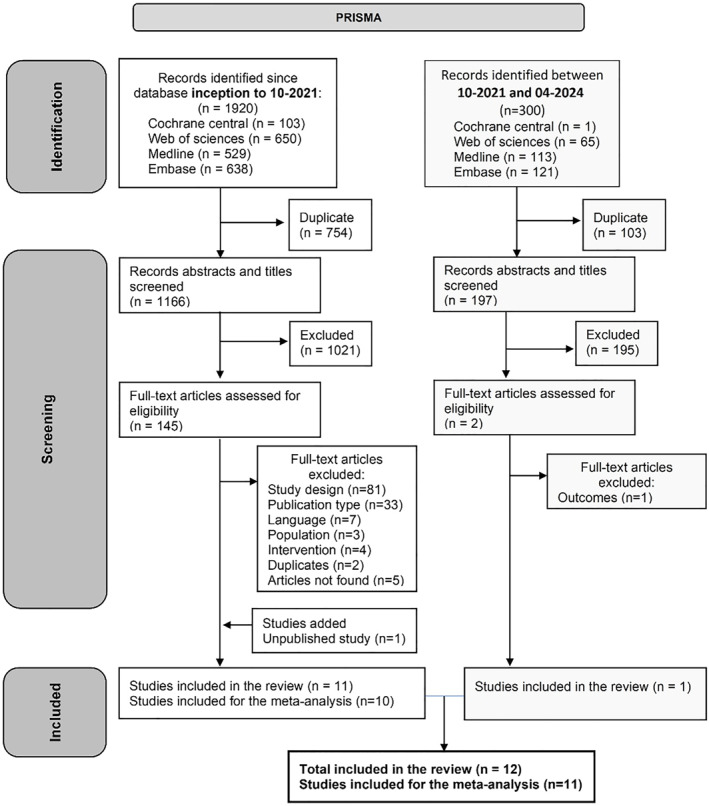
PRISMA flow diagram.

Databases were searched up to April 2024 and revealed 2200 studies of which 145 were assessed in full‐text (Figure 1) and eleven met eligibility criteria. Citation tracking led to the identification of one unpublished study on clinicaltrials.gov that met all the inclusion criteria. However, from the 12 studies included in the review, one (Yavuz et al. 2012) was excluded from the meta‐analyses due to outlier results (Figure 1).

The included studies represented 1125 participants (mean age 63 years, 44% of women). The severity of knee OA was mild to moderate in four studies (Conaghan, Hunter, et al. 2018; McAlindon et al. 2017; Mendes, Natour, Nunes‐Tamashiro, Toffolo, Rosenfeld, and Furtado 2019a; Nunes‐Tamashiro et al. 2022), mild to severe in three studies(Lindsley 2000; Ravaud et al. 1999; Yavuz et al. 2012), and not mentioned in five studies(Chao et al. 2010; Friedman and Moore, 1980; Gaffney et al. 1995; Shrestha et al. 2018; Young et al. 2001). Pain was mainly assessed with VAS (Chao et al. 2010; Gaffney et al. 1995; McAlindon et al. 2017; Mendes, Natour, Nunes‐Tamashiro, Toffolo, Rosenfeld, and Furtado 2019a; Nunes‐Tamashiro et al. 2022; Ravaud et al. 1999; Shrestha et al. 2018; Yavuz et al. 2012) and WOMAC (Lindsley 2000; Young et al. 2001).

Associated interventions were present in 6 studies, with knee aspiration before injection in six studies (Friedman and Moore, 1980; Gaffney et al. 1995; McAlindon et al. 2017; Mendes, Natour, Nunes‐Tamashiro, Toffolo, Rosenfeld, and Furtado 2019a; Nunes‐Tamashiro et al. 2022; Ravaud et al. 1999), physiotherapy programme in one study (Shrestha et al. 2018), and oral medication allowed in five studies (Friedman and Moore, 19800; McAlindon et al. 2017; Nunes‐Tamashiro et al. 2022; Ravaud et al. 1999; Shrestha et al. 2018) (Table 1).

**TABLE 1:** Summary of included studies.

Author(s), year	Quality^a^	Population	Inclusion criteria	Intervention	Results
Chao et al. (2010)	4/5	I: *n* = 34 C: *n* = 33 age 64 sex(w%) 3 s% 35	ACR criteria and x‐ray in the last 12 months	Landmark injection, I: 40 mg TA 1 mL C: NS 1 mL No other intervention	Reduction significantly better in TA group for VAS and WOMAC pain subscale at 4 weeks, not at 12 weeks.
Conaghan, Hunter, et al. (2018)	5/5	I1: *n* = 161 I2: *n* = 162 C: *n* = 163 age 62 sex(w%)61 s% nm	ACR criteria, K‐L 2 or 3, age > 40, pain > 15 days in the last month, ADP of ≥ 5 and ≤ 9 for ≥ 5 days	Landmark injection I1: 32 mg FX006 5 mL I2: 40 mg TA 1 mL C: NS 5 mL No other intervention	The main objective was to compare FX006 to NS and TA. FX006 was superior to NS for ADP, WOMAC, and KOOS‐QOL up to 12 weeks. No significant difference between FX006 and TA at any time.
Friedman and Moore, (1980)	4/5	I: *n* = 17 C: *n* = 17 age 42‐77 sex(w%) nm s% nm	OA finding in physical exam, no sign of INF, age > 40	Landmark injection, I: TH 20 mg C: NS Aspiration done, allowed to continue previous medication	NPRS reduction was significantly better for TH than NS at 1 week. No significant difference at 4, 6, and 8 weeks.
Gaffney et al. (1995)	1/5	I: *n* = 42 C: *n* = 42 age 67 sex(w%)29 s% 38	Clinical and radiologic evidence of knee OA	Landmark injection, I: 20 mg TH 1 mL C: NS 1 mL Aspiration done	Pain reduction on VAS was significant in both TH and NS at 1 and 6 weeks. TH was significantly superior to NS at 1 week only.
Lindsley (2000)	3/5	I1: *n* = 8 I2: *n* = 4 C: *n* = 4 age nm sex(w%)100 s% 100	Age 35‐85, VAS ≥ 30, mild to moderate OA changes on x‐ray	Injection NM, I1: IFX 100 mg I2: 80 mg MP C: NS Other intervention NM	WOMAC change at 12 weeks: −25.9 ± 18.1 in infliximab, 2.0 ± 5.8 in MP, and −9.3 ± 27.2 in NS
McAlindon et al. (2017)	5/5	I: *n* = 70 C: *n* = 70 age 58 sex(w%) 54 s% 100	ARC criteria, K‐L 2‐3, age > 45, WOMAC 2‐8, United States evidence of synovitis	US‐guided injection, I: 40 mg TA 1 mL C: NS 1 mL Aspiration done, PRN acetaminophen	This study evaluated the effect of regular tri‐monthly injections on pain (VAS), function (WOMAC, 20mwt, CST), and cartilage in a 2‐year follow‐up. No difference in pain or function at the 2 years point with significant loss of cartilage in the TA group.
Mendes, Natour, Nunes‐Tamashiro, Toffolo, Rosenfeld, and Furtado (2019)	5/5	I1: *n* = 35 I2: *n* = 35 C: *n* = 35 age 64 sex(w%) 91 s% nm	Age > 50, K‐L 2‐3, VAS 3‐8, pain > 6 months	Landmark injection, I1: 100 IU 2 mL I2: 40 mg TH 2 mL C: NS 2 mL Aspiration done	Pain reduction was significantly better for TH compared to NS and BTA at 4 and 8 weeks for movement VAS. No significant difference for WOMAC, 6MWT, and SF‐36 for all groups.
Nunes‐Tamashiro et al. (2022)	5/5	I1: *n* = 33 I2: *n* = 34 C: *n* = 33 age 67 sex(F) 90 s% nm	Age > 40 < 85, K‐L 2‐3, bilateral OA of the knee, pain > 3 months, VAS > 4 < 8	Landmark injection, I1: 40 mg TA I2: PRP C: NS 2 mL	No significant difference between the groups on pain on movement or at rest at 4, 8, 12 and 52 weeks. Improvements were significant in both groups at every assessment times compared to baseline.
Shrestha et al. (2018)	5/5	I: *n* = 57 C: *n* = 70 age 67 sex(w%) 62 s% nm	ACR criteria	Landmark injection, I: 40 mg TA C: NS Aceclofenac first week and physiotherapy	Effect significantly superior in TA compared to NS at 2 and 6 weeks for VAS, and 2, 6, and 12 weeks for KOOS‐PS. VAS improvement from baseline for TA was significant only at 2 and 6 weeks.
Ravaud et al. (1999)	3/5	I1: *n* = 25 I2: *n* = 21 I3: *n* = 24 C: *n* = 28 age 65 sex(w%) 67 s% 47	ACR criteria, K‐L ≥ 2, VAS ≥ 40	Landmark injection, I1: 3,75 mg CZ 1.5 mL I2: JL + NS I3: JL + CZ 3,75 mg C: NS 1.5 mL Aspiration done, PRN acetaminophen and NSAID	Additive effect of joint lavage and CZ. Pain reduction on VAS was significant only at 1 and 4 weeks for CZ, with a peak effect at 1 week.
Yavuz et al. (2012)	0/5	I1: *n* = 30 I2: *n* = 30 I3: *n* = 30 C: *n* = 30 age 60 sex(w%) 63 s% nm	55–75 years old, ACR criteria, K‐L ≥ 2, age 55‐75, VAS ≥ 5	Landmark injection, I1: 3 mg BM 1 mL I2: 40 mg MP 1 mL I3: 40 mg TA 1 mL C: NS 1 mL No other intervention	Improvement in VAS and LFI was significantly superior in all groups compared to NS up to 12 weeks. MP was significantly superior to BM and MP in VAS at 1 and 3 weeks.
Young et al. (2001)	3/5	I: *n* = 20 C: *n* = 20 age 67 sex(w%) 40 s% nm	Arthroscopic grading	Injection during arthroscopic biopsy I: MP 120 mg, mL NM C: NS	The main outcome was the effect on chemokines expressions. Clinical results: significant moderate reduction WOMAC at 12 weeks in favour of MP, no difference in walking time.

Abbreviations: 20 mwt = 20 m walk test, 6 MWT = 6 minute‐walk‐test, ACR = American College of Rheumatology, ADP = average daily pain, Age = mean age, BM = betamethazone disodium phosphate, BTA = botulinum toxin A, C = control group, CST = chair stand test, CZ = corticazol, i = intervention group, IFX = infliximab, INF = inflammation, JL = joint lavage, K‐L = Kellgren‐Lawrence Radiologic Classification, KOOS‐PS = Knee injury and Osteoarthritis Outcome Score‐Physical Function Shortform, KOOS‐QOL = Knee injury and Osteoarthritis Outcome Score – quality of life, LFI = Lequesne functional index, LFI = Lequesne functional index, MP = methylprednisolone, *n* = number of participants included in the study, NM = not mentioned, NPRS = numeric pain rating scale, NS = normal saline, OA = osteoarthritis, PRP = platelet rish plasma, S% = synovitis percentage, S.D. = study duration, ST‐36 = The Short‐Form Health Survey, TA = triamcinolone acetonide, TH = triamcinolone acetonide, US = ultrasound, VAS = visual analog scale, Sex (W%) = women percentage, WOMAC = Western Ontario and McMaster Universities Arthritis Index, y = years.

^a^ Jadad Score.

We requested additional data from six authors by email. Two replied, but only one was able to provide information relevant to this review. Results were therefore extrapolated by imputing the coefficient of correlation in eight studies (Conaghan, Hunter, et al. 2018; McAlindon et al. 2017; Mendes, Natour, Nunes‐Tamashiro, Toffolo, Rosenfeld, and Furtado 2019a; Nunes‐Tamashiro et al. 2022; Ravaud et al. 1999; Shrestha et al. 2018; Yavuz et al. 2012; Young et al. 2001) and extracted from the graphs in two studies (McAlindon et al. 2017; Young et al. 2001) (see Supporting Information S1: appendix 3).

### 3.2 Quality of the Studies

The mean Jadad score was 3.6/5. The most common flaws were missing information on randomisation (Chao et al. 2010; Gaffney et al. 1995; Lindsley 2000; Ravaud et al. 1999; Yavuz et al. 2012; Young et al. 2001), blinding (Gaffney et al. 1995; Lindsley 2000; Ravaud et al. 1999; Yavuz et al. 2012), and dropouts (Friedman and Moore, 1980; Gaffney et al. 1995; Yavuz et al. 2012; Young et al. 2001). Jadad's total scores can be found in Table 1 and score details can be found in Supporting Information S1: appendix 4.

### 3.3 Quality of the Evidence

The quality of evidence varied from moderate to very low (Table 2). The main reasons for downgrading were the risk of bias, indirectness, and imprecision (details in Supporting Information S1: appendix 5).

**TABLE 2:** GRADE assessment—Evidence on the effect of different doses of IACI on pain reduction compared to normal saline, and between‐dose comparison analysis.

	Pain reduction against placebo			Influence of the dose		
	Low dose	Usual dose	High dose	VST	ST	MT
Risk of bias	−1	0	−1	0	0	0
Inconsistency	0	−1	−1	0	−1	−1
Indirectness	0	0	0	−2	−2	−2
Imprecision	−1	0	−1	−1	0	−1
Publication bias	0	0	0	0	0	0
Upgrade	None	None	None	None	None	None
Overall GRADE	Low	Moderate	Very low	Very low	Very low	Very low

Abbreviations: MT = middle term, ST = short term, VST = very short term.

### 3.4 Meta‐Analyses

The meta‐analysis included 1005 participants, due to the exclusion of (Yavuz et al. 2012).

1. The effect of different doses of corticosteroid on pain reduction compared to normal saline

**FIGURE 2:**
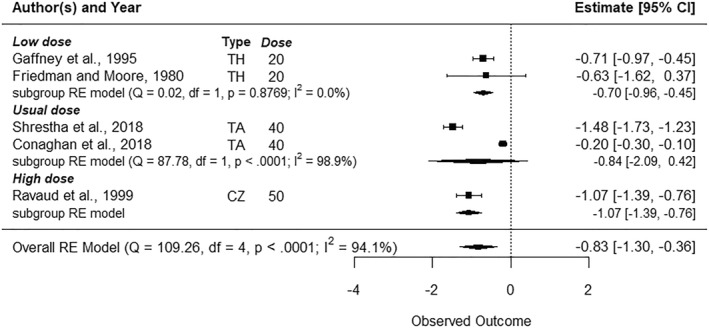
Forest plots—Effect of intra‐articular corticosteroid injections on pain reduction in the very short term (1–3 weeks) when compared to normal saline. BM = betamethasone, CZ = cortivazol, df = degree of freedom, Dose = dose equivalence of 40 mg of methylprednisolone, MP = methylprednisolone, Q = *Q*‐value, RE model = random effect model, TA = triamcinolone, TH = triamcinolone hexacetonide, Type = corticosteroid type.

**FIGURE 3:**
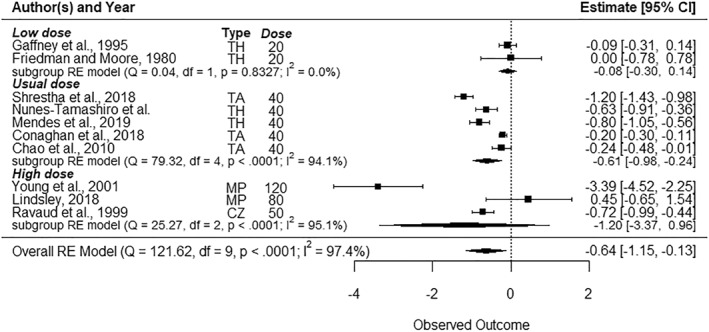
Forest plots—Effect of intra‐articular corticosteroid injections on pain reduction in the short term (4–8 weeks) when compared to normal saline. BM = betamethasone, CZ = cortivazol, df = degree of freedom, Dose = dose equivalence of 40 mg of methylprednisolone, MP = methylprednisolone, Q = *Q*‐value, RE model = random effect model, TA = triamcinolone, TH = triamcinolone hexacetonide, Type = corticosteroid type.

**FIGURE 4:**
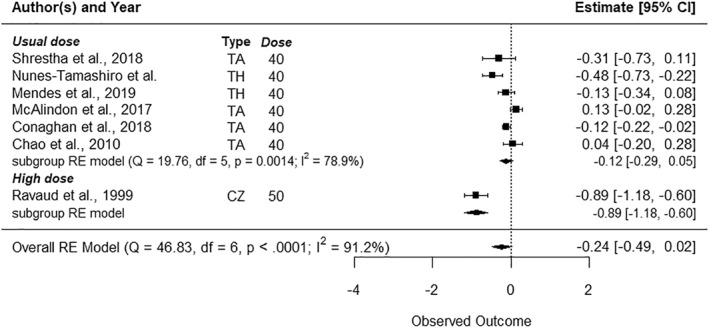
Forest plots—Effect of intra‐articular corticosteroid injections on pain reduction in the middle term (10–16 weeks) when compared to normal saline. BM = betamethasone, CZ = cortivazol, df = degree of freedom, Dose = dose equivalence of 40 mg of methylprednisolone, MP = methylprednisolone, Q = *Q*‐value, RE model = random effect model, TA = triamcinolone, TH = triamcinolone hexacetonide, Type = corticosteroid type.

Five studies included VST follow‐ups (Figure 2) (Friedman and Moore, 1980; Gaffney et al. 1995; Ravaud et al. 1999; Shrestha et al. 2018; Yavuz et al. 2012), ten included ST follow‐ups (Figure 3) (Chao et al. 2010; Friedman and Moore, 1980; Gaffney et al. 1995; Lindsley 2000; Mendes et al. 2019a, 2019b; Ravaud et al. 1999; Shrestha et al. 2018; Yavuz et al. 2012; Young et al. 2001), and seven included MT follow‐up (Figure 4) (Chao et al. 2010; McAlindon et al. 2017; Mendes, Natour, Nunes‐Tamashiro, Toffolo, Rosenfeld, and Furtado 2019a; Nunes‐Tamashiro et al. 2022; Ravaud et al. 1999; Shrestha et al. 2018; Yavuz et al. 2012). The funnel plots can be found in Supporting Information S1: appendix 6.

Low doses were evaluated by two studies(Friedman and Moore, 1980; Gaffney et al. 1995) in the VST and ST (*n* = 118). The effect of low dose on pain reduction was statistically significant in the VST group, with a moderate effet size (SMD = −0.70 [95% CI −0.96 to −0.45], *p* < 0.01). It was not significant in the ST. There was no significant statistical heterogeneity between studies at VST (*I* ^2^ = 0%) and ST (*I* ^2^ = 0%).

The usual dose was evaluated by six studies (Chao et al. 2010; Conaghan, Hunter, et al. 2018; McAlindon et al. 2017; Mendes, Natour, Nunes‐Tamashiro, Toffolo, Rosenfeld, and Furtado 2019a; Nunes‐Tamashiro et al. 2022; Shrestha et al. 2018), in the VST, ST and MT (*n* = 785). The usual dose had a significant effect on pain reduction in the ST, with a moderate effect size (SMD = −0.61 [95% CI −0.98 to −0.24], *p* = 0.01). No significant effect was found in the VST and MT. Heterogeneity between studies was very high for VST (*I* ^2^ = 99%), ST (*I* ^2^ = 94.1%), and MT (*I* ^2^ = 78.9%).

Finally, high doses were evaluated by three studies(Lindsley 2000; Ravaud et al. 1999; Young et al. 2001), in the VST, ST and MT (*n* = 102). Based on one study(Ravaud et al. 1999), there was a significant high effect size of high dose on pain reduction in the VST (SMD = −1.07 [95% CI −1.39 to 0.76], *p* < 0.01) and MT (SMD = −0.89 [95% CI −1.18 to −0.60], *p* < 0.01). No significant effect was found at ST. Heterogeneity between studies was high for ST (*I* ^2^ = 95%) and was not measurable for VST and MT.

2 The influence of dosage on pain reduction

Multivariate analysis showed that dose had a significant effect on pain reduction, with higher dose being associated with greater pain reduction in the ST (ES = −0.35 [95% CI: −0.52 to −0.18], *p* < 0.01) and in the MT (ES = −0.80 [95% CI: −1.10 to −0.50], *p* < 0.01), although significant residual heterogeneity was present for all time frame (*p* < 0.01). No effect of dosage was identified in the VST (*p* = 0.37).

### 3.5 Adverse Effects

Adverse effects were present in 29/525 (5.5%) of the participants receiving IACI, and 23/368 (6.3%) receiving saline injection. No serious adverse effects were reported in any study. A detailed report of adverse effects can be found in the Table 3.

**TABLE 3:** Adverse effects reported in included studies.

Author(s), year	Group, *n*	Adverse effects description and percentage of patients
Chao et al. (2010)	Total, 67	Not reported
Conaghan, Cohen, et al. (2018a)	Total, 323	A total of seven adverse effects were considered possibly related to the injected agent
	Active, 161	Four (2.5%) cases without details and five cases of radiographic OA progression
	Saline, 162	Three (1.9%) cases without details and six cases of radiographic OA progression
Friedman and Moore (1980)	Total, 34	
	Active, 17	Four (23.5%) cases of post‐injection flares
	Saline, 17	Five (29.4%) cases of post‐injection flares
Gaffney et al. (1995)	Total, 84	Not reported
Lindsley (2000)	Total, 8	No serious adverse effects reported
	Active, 4	One (25%) case of local pain post‐injection, one (25%) case of local rash
	Saline, 4	One (25%) case of local pain after the biopsy, one (25%) case of knee sprain
McAlindon et al. (2017)	Total, 140	After 2 years of repeated injection, 115 side effects were reported, with only 8 considered related to the injections. The study showed a significant narrowing of the cartilage on the steroid group compared to the saline. There was no difference in HbA1c and no case of osteonecrosis
	Active, 70	Four (5.7%) local pain post‐injection, one (1.4%) flushing reaction, and two (2.9%) cases of hypertension
	Saline, 70	Two (2.9%) local pain post‐injection, one (1.4%) case of hypertension and one (1.4%) case of cellulitis
Mendes, Natour, Nunes‐Tamashiro, Toffolo, Rosenfeld, and Furtado (2019)	Total, 70	No adverse effects during the study
Nunes‐Tamashiro et al. (2022)	Total, 66	No adverse effects during the study
	Active, 33	No adverse effects during the study
	Saline, 33	No adverse effects during the study
Ravaud et al. (1999)	Total, 53	One flare up post‐injection, group non specified
Shrestha et al. (2018)	Total, 117	No serious adverse effects
	Active, 57	2 cases of pain during procedure (8%)
	Saline, 60	5 cases of pain during procedure (18%)
Yavuz et al. (2012)	Total, 120	No adverse effects reported during the study
Young et al. (2001)	Total, 41	Not reported

Abbreviation: OA = osteoarthritis.

For low doses, adverse effects were reported in 4% (*n* = 107) of the participants receiving IACI and 10% (*n* = 46) receiving saline. For the usual dose, adverse effects were reported in 5% (*n* = 389) participants receiving IACI and 4% (*n* = 330) receiving saline injection. For high doses, adverse effects were reported in 14% (*n* = 29) participants receiving IACI and 19% (*n* = 32) receiving saline injections.

## 4 Discussion

This systematic review and meta‐analysis aimed to compare the efficacy of IACI on pain in knee OA to saline and the influence of dosing. The results showed that the efficacy of low doses was greater than normal saline at VST, that the usual dose was more efficacious than saline in the ST, and that a high dose was more efficacious in the MT. It revealed that the maximal effect for all doses was in the VST, but only the effects of high doses lasted up to MT. These findings were confirmed by the multivariate analyses that showed that dose significantly influenced pain reduction at ST and MT, but not at VST. The quality of evidence was moderate to low.

Altogether, these findings seem in line with studies directly comparing different doses, which reported no difference in the peak effect (VST), but a longer effect in the group receiving the higher IACI dose (Conaghan, Cohen, et al. 2018; Pyne et al. 2004). However, other studies showed no difference when directly comparing different dosages (Bias et al. 2001; Bodick et al. 2015; Popma et al. 2015; Robinson et al. 2007; Utamawatin et al. 2023), limiting the interpretation of these findings.

### 4.1 IACI Safety

Few non‐serious adverse effects were reported but were similar between the IACS and normal saline groups. The prevalence of adverse effects obtained in the present study was lower than the one reported in other meta‐analysis (i.e., up to 13%–19% for IACS and 15%–27% for control groups) (Jüni et al. 2015; Saltychev et al. 2020). This difference could be explained by different factors: (1) the exclusion of lidocaine, which can be associated with side effects, (2) the exclusion of patients at high risk in some studies (e.g. uncontrolled diabetes and hypertension) (Conaghan, Hunter, et al. 2018; McAlindon et al. 2017; Yavuz et al. 2012), and (3) lack of data information about adverse effects(Chao et al. 2010; Gaffney et al. 1995; Young et al. 2001). Finally, the percentage of adverse effects seems to increase with the IACI dose, but it also increases in the corresponding normal saline group, suggesting an undetermined underlying factor not related to the IACI dose.

### 4.2 Strengths of the Study

First, this systematic review used an extensive search protocol leading to a large number of screened articles. Second, we excluded the combination of corticosteroid with any other product, including local anaesthetic, as a previous study showed that injection of such products could lead to a lasting effect over 3 months (Eker et al. 2017). As a result, the effect of IACI on pain could be more precisely evaluated and allow indirect comparison. This led, to our knowledge, to the first systematic review showing an effect of dose on pain reduction in IACI for knee OA.

### 4.3 Limitations of the Study

First, no protocol was previously published, and the review was not registered.

Second, indirect comparison of subgroups was used because there were not enough studies directly comparing different doses in knee OA, as noted in a previous review (Cushman et al. 2018). Moreover, it was not possible to perform a meta‐regression due to the small dispersion of the studied doses. Therefore, a dose subgroup comparison through multivariate analysis was performed.

Third, the strict inclusion criteria chosen for the injectate, allowing for an indirect subgroup comparison, led to a small number of included studies, especially in low and high doses, with only two (Friedman and Moore, 1980; Gaffney et al. 1995) and three (Lindsley 2000; Ravaud et al. 1999; Young et al. 2001) studies, respectively. This was accentuated by the exclusion of (Yavuz et al. 2012), that was included in the review, but excluded from the meta‐analysis because it led to outlier results, which was also noticed in a previous review (Saltychev et al. 2020). The outlier results might come from the very high risk of bias of the study (Jadad scale of 0/5).

Fourth, the statistical power was limited due to small samples (Conaghan, Hunter, et al. 2018; McAlindon et al. 2017; Shrestha et al. 2018), and the extrapolation of missing data (Conaghan, Hunter, et al. 2018; McAlindon et al. 2017; Mendes, Natour, Nunes‐Tamashiro, Toffolo, Rosenfeld, and Furtado 2019a; Nunes‐Tamashiro et al. 2022; Ravaud et al. 1999; Shrestha et al. 2018; Yavuz et al. 2012; Young et al. 2001).

Fifth, heterogeneity between studies was generally high, which limits the generalisation of the findings. This high heterogeneity could explain why the usual dose was not statistically significant in the VST, while both studies showed the superiority of corticosteroids over normal saline. There are potential explanations for this heterogeneity, including the variety of IACI products(Cole and Schumacher 2005), study quality, OA diagnostic tools, and pain measurement tools used in the studies, along with the varying time assessments, and the guidance technique used (anatomical landmarks could lead to 4.5%–22.7% of extra‐articular injection (Fang et al. 2021)). No specific sensitivity analysis was performed to evaluate their effect on heterogeneity.

## 5 Conclusion

Based on very low quality of evidence, results suggest that higher doses of IACI might prolong pain reduction in the middle term. However, the dose does not influence the peak effect of IACI in the first weeks. This has important clinical implications: if the clinical objective is rapid pain relief, as used in diagnostic injections and in acute pain flare up, lower doses should be considered. If the objective is to have a long‐lasting effect, higher doses might be considered. However, further studies are needed to confirm the results.

## Author Contributions

M.A.L. and F.D. screened the articles, extracted the data, performed quality assessments and rated the quality of evidence. M.A.L. performed the statistical analysis and wrote the manuscript. All authors contributed to the protocol of this review and revised the manuscript.

## Ethics Statement

The authors have nothing to report.

## Consent

The authors have nothing to report.

## Conflicts of Interest

The authors declare no conflicts of interest.

## Supporting information

Supporting Information S1

## Data Availability

All data used in the analysis are presented in this article and its supplementary files.
