# Supplementary material for: The Effect of Corticosteroid Doses on Pain in Knee Osteoarthritis: A Systematic Review and Meta‐Analysis
Source: Musculoskeletal Care. 2025 May 19;23(2):e70121. doi: 10.1002/msc.70121 (PMC12089009; doi:10.1002/msc.70121)
Supplement: Supplementary file 1 — Supporting Information S1 [file MSC-23-e70121-s001.docx]

**Supplementary files**

**The effect of corticosteroid doses on pain in knee osteoarthritis: a systematic review and meta-analysis**

Marc-Antoine Lafrenaye-Dugas, MD^1,6^, Frédérique Dupuis, M.Pht, M.Sc^2,3^, Valérie Bélanger, MD^1,3^, Marie-Michèle Briand MD, M.Sc^2,3,4,5^

^1^Department of Physical Medicine and Rehabilitation (Physiatry), Centre Hospitalier Universitaire de Québec – Université Laval, Quebec City, QC, Canada.

^2^ Faculty of Medicine, Université Laval, Quebec City, QC, Canada.

^3^Center for Interdisciplinary Research in Rehabilitation and Social Integration of Quebec, Quebec City, QC, Canada.

^4^Center of Research of the Hôpital du Sacré-Coeur de Montreal, CIUSSS du Nord-de-l’Île-de-Montreal, Montreal, QC, Canada.

^5^Faculty of Medicine, Université de Montréal, Montréal, QC, Canada

^6^ Faculté de Médecine de l’Université de Sherbrooke, Sherbrooke, QC, Canada

**APPENDIX 1.**

**FULL SEARCH STRATEGY.**

| **MEDLINE** |
| --- |
| "(""Adrenal Cortex Hormones""[MeSH Terms] OR ""Hydroxycorticosteroids""[MeSH Terms] OR (""adrenal cortex hormone*""[Title/Abstract] OR ""corticosteroid*""[Title/Abstract] OR ""corticoid*""[Title/Abstract] OR ""steroid*""[Title/Abstract] OR ""hydroxycorticosteroid*""[Title/Abstract] OR ""triamcinolone*""[Title/Abstract] OR ""methylprednisolone*""[Title/Abstract] OR ""dexamethasone*""[Title/Abstract] OR ""betamethasone*""[Title/Abstract] OR ""prednisone*""[Title/Abstract] OR ""prednisolone*""[Title/Abstract])) AND  (""injections, intra articular""[MeSH Terms] OR (""intraarticular inject*""[Title/Abstract] OR ""intra articular inject*""[Title/Abstract] OR ""intra articular inject*""[Title/Abstract] OR ""intra-articular""[Title/Abstract] OR ""intraarticular""[Title/Abstract] OR ""intra-articular""[Title/Abstract])) AND  (""Osteoarthritis""[MeSH Terms] OR (""osteoarth*""[Title/Abstract] OR ""degenerative arthrit*""[Title/Abstract] OR ""Arthrosis""[Title/Abstract] OR ""Arthroses""[Title/Abstract])) AND  (""Knee Joint""[MeSH Terms] OR (""knee joint*""[Title/Abstract] OR ""superior tibiofibular joint*""[Title/Abstract] OR ""knee""[Title/Abstract])) AND  ((""randomized controlled trial""[Publication Type] OR ""controlled clinical trial""[Publication Type] OR ""randomized""[Title/Abstract] OR ""placebo""[Title/Abstract] OR ""drug therapy""[MeSH Subheading] OR ""randomly""[Title/Abstract] OR ""trial""[Title/Abstract] OR ""groups""[Title/Abstract]) NOT (""animals""[MeSH Terms] NOT ""humans""[MeSH Terms]))" |
| **EMBASE** |
| (('corticosteroid'/exp OR ('adrenal cortex hormone*':ti,ab,kw OR corticosteroid*:ti,ab,kw OR corticoid*:ti,ab,kw OR steroid*:ti,ab,kw OR hydroxycorticosteroid*:ti,ab,kw OR triamcinolone*:ti,ab,kw OR methylprednisolone*:ti,ab,kw OR dexamethasone*:ti,ab,kw OR betamethasone*:ti,ab,kw OR prednisone*:ti,ab,kw OR prednisolone*:ti,ab,kw)) AND  ('intraarticular drug administration'/exp OR ('intraarticular inject*':ti,ab,kw OR 'intra-articular inject*':ti,ab,kw OR 'intra articular inject*':ti,ab,kw OR intraarticular:ti,ab,kw OR 'intra articular':ti,ab,kw OR 'intra-articular administration':ti,ab,kw OR 'intra-articular delivery':ti,ab,kw OR 'intra-articular drug administration':ti,ab,kw OR 'intra-articular treatment':ti,ab,kw OR 'intraarticular administration':ti,ab,kw OR 'intraarticular delivery':ti,ab,kw OR 'intraarticular drug administration':ti,ab,kw OR 'intraarticular treatment':ti,ab,kw)) AND  ('osteoarthritis'/exp OR (osteoarth*:ti,ab,kw OR 'degenerative arthrit*':ti,ab,kw OR arthrosis:ti,ab,kw OR arthroses:ti,ab,kw OR 'degenerative joint disease':ti,ab,kw OR 'osteo arthr*':ti,ab,kw)) AND  ('knee'/exp OR ('knee joint*':ti,ab,kw OR 'superior tibiofibular joint*':ti,ab,kw OR knee:ti,ab,kw))) AND  ('crossover procedure':de,ti,ab,kw OR 'double-blind procedure':de,ti,ab,kw OR 'randomized controlled trial':de,ti,ab,kw OR 'single-blind procedure':de,ti,ab,kw OR random*:ti,ab,kw OR factorial*:ti,ab,kw OR crossover*:ti,ab,kw OR ((cross NEXT/1 over*):ti,ab,kw) OR placebo*:ti,ab,kw OR ((doubl* NEAR/1 blind*):ti,ab,kw) OR ((singl* NEAR/1 blind*):ti,ab,kw) OR assign*:ti,ab,kw OR allocat*:ti,ab,kw OR volunteer*:ti,ab,kw) |
| **Cochrane centrale** |
| MeSH descriptor: [Adrenal Cortex Hormones] explode all trees OR MeSH descriptor: [Hydroxycorticosteroids] explode all trees OR (“Adrenal Cortex Hormone*” OR Corticosteroid* OR Corticoid* OR Steroid* OR Hydroxycorticosteroid* OR Triamcinolone* OR methylprednisolone* OR Dexamethasone* OR Betamethasone* OR Prednisone* OR Prednisolone*):ti,ab,kw AND  (“Intraarticular inject*” OR “intra-articular inject*” OR “intra articular inject*” OR intra-articular OR intraarticular OR “intra articular” OR “intra-articular administration” OR “intra-articular delivery” OR “intra-articular drug administration” OR “intra-articular treatment” OR “intraarticular administration” OR “intraarticular delivery” OR “intraarticular drug administration” OR “intraarticular treatment”):ti,ab,kw AND  MeSH descriptor: [Osteoarthritis] explode all trees OR (Osteoarth* or “degenerative arthrit*” or Arthrosis or Arthroses or “degenerative joint disease” or osteo-arthr*):ti,ab,kw AND  MeSH descriptor: [Knee Joint] explode all trees OR (“knee joint*” or “Superior Tibiofibular Joint*” or knee):ti,ab,kw |
| **Web of Science** |
| (“Adrenal Cortex Hormone*” OR Corticosteroid* OR Corticoid* OR Steroid* OR Hydroxycorticosteroid* OR Triamcinolone* OR methylprednisolone* OR Dexamethasone* OR Betamethasone* OR Prednisone* OR Prednisolone*) AND  (“Intraarticular inject*” OR “intra-articular inject*” OR “intra articular inject*” OR intra-articular OR intraarticular OR “intra articular” OR “intra-articular administration” OR “intra-articular delivery” OR “intra-articular drug administration” OR “intra-articular treatment” OR “intraarticular administration” OR “intraarticular delivery” OR “intraarticular drug administration” OR “intraarticular treatment”) AND  (Osteoarth* or “degenerative arthrit*” or Arthrosis or Arthroses or “degenerative joint disease” or osteo-arthr*) AND  (“knee joint*” or “Superior Tibiofibular Joint*” or knee) AND  (“randomized controlled trial*” OR “controlled clinical trial” OR “placebo” or “random*” or trial* or group* or “double-blind*” or “double blind*” or “single-blind*” or “single blind*”) |

**APPENDIX 2.**

**DATA EXTRACTION FORM.**

| **Article title** |  | |
| --- | --- | --- |
| **Authors** |  | |
| **Country** |  | |
| **Objective** |  | |
| **Population** | | |
| Sample size, N | |  |
| Age (mean, SD) | |  |
| Women, n (%) | |  |
| Synovitis, n (%) | |  |
| Recruitment strategy | |  |
| Randomization | |  |
| Inclusion criteria (OA severity) | |  |
| Exclusion criteria | |  |
| Other population characteristics (ex. comorbidity) | |  |
| **Methods** | | |
| Type of corticosteroid | |  |
| Dose (mg) | |  |
| Infiltration technique (guided?) | |  |
| Aspiration done? | |  |
| Number of infiltration and administration frequency | |  |
| Total study duration | |  |
| Follow-up assessment frequency (ex. 2 weeks, 3 months, etc.) | |  |
| Control group and n | |  |
| Other interventions  (ex. Physical therapy and short description) | |  |
| Outcomes (variables of interest) | |  |
| Type of assessment (questionnaire, VAS, etc.) | |  |
| Other outcomes of interest (ex. Radiological assessment, ROM, strength, etc.) | |  |
| Data analysis | |  |
| Blinding (evaluator, pts [yes, no]) | |  |
| **Results** | | |
| Dropout rate | |  |
| Results: include all information (CI, mean, …)  Type of analysis and p-values | | |
| Baseline normal saline | |  |
| Baseline steroid | |  |
| T1 normal saline | |  |
| T1 steroid | |  |
| T2 normal saline | |  |
| T2 steroid | |  |
| T3 normal saline | |  |
| T3 steroid | |  |
| Results on other outcomes | |  |
| Adverse effect | |  |
| Short description | |  |
| Pertinent discussion points | |  |
| Study quality (JADAD) | | |
| Was the study described as randomized? | | |
| Was the method used to generate the sequence of randomization described and appropriate? | | |
| Was the study described as double blind? | | |
| Was the method of double blinding described and appropriate? | | |
| Was there a description of withdrawals and dropouts? | | |
| Deduct one point if the method used to generate randomization was described and it was inappropriate. | | |
| Deduct one point if the study was described as double blind but the method of blinding was inappropriate. | | |
| Total | | |

**APPENDIX 3.**

**DETAILED RESULTS SYNTHESIS**

1. Estimation of the results when standard deviation of change was not reported

When standard deviations of changes were not reported, they were estimated by the calculator with this formula: SD.change = sqrt(SD.pre + SD.post - 2 COVAR.pre.post). Rarely reported, covariances were derived from correlation coefficients drawn from the scientific literature via the reliability test for these scales (ICC=0.96-0.98 for VAS in knee OA^1^ and ICC=0.71-0.95 for WOMAC) ^2, 3^. Conservatively, we used 0.9 for studies using VAS and 0.7 for studies using WOMAC.

1. Estimation of the results using graphs.

When means or SD were missing in the manuscript and good quality graphs were present, we extracted the results from the graphs using the pixels as reference. Graphs were imported in paint to allow to convert the measures in pixels. The number of pixels were than used in the same formula previously described (SD.change = sqrt[SD.pre + SD.post - 2 COVAR.pre.post]). The method was already confirmed in a preview study.^4^

1. Multiple results in a time frame
2. When multiple data were available for the same time frame, we used a mean of all the available data in the time frame (i.e., if data were available at 5 and 8 weeks, the mean of the two results was used for the short term (4-8 weeks)).
3. Results outside of our time frame windows (1-3 weeks, 4-8 weeks, and 10-16 weeks) were not considered.
4. References

1. Alghadir AH, Anwer S, Iqbal A, Iqbal ZA. Test-retest reliability, validity, and minimum detectable change of visual analog, numerical rating, and verbal rating scales for measurement of osteoarthritic knee pain. Journal of pain research. 2018;11:851-6.

2. Kuptniratsaikul V, Rattanachaiyanont M. Validation of a modified Thai version of the Western Ontario and McMaster (WOMAC) osteoarthritis index for knee osteoarthritis. Clinical Rheumatology. 2007;26(10):1641-5.

3. Basaran S, Guzel R, Seydaoglu G, Guler-Uysal F. Validity, reliability, and comparison of the WOMAC osteoarthritis index and Lequesne algofunctional index in Turkish patients with hip or knee osteoarthritis. Clinical Rheumatology. 2010;29(7):749-56.

4. Van der Mierden S, Spineli LM, Talbot SR, Yiannakou C, Zentrich E, Weegh N, et al. Extracting data from graphs: A case-study on animal research with implications for meta-analyses. Research Synthesis Methods.n/a(n/a).

**APPENDIX 4.**

**DETAILED JADAD QUALITY SCORE**

| **Author(s),** year | Randomized | Randomization described? | Double blinded | Blinding method described? | Withdrawals and dropouts described? | Inappropriate randomization method* | Inappropriate blinding method^Ʈ^ | Total |
| --- | --- | --- | --- | --- | --- | --- | --- | --- |
| **Yavuz et al.,** 2012^40^ | Yes | No | No | No | No | -1 | 0 | 0 |
| **Gaffney et al.,** 1995^51^ | Yes | No | Yes | No | No | 0 | -1 | 1 |
| **Lindsley,** 2018^46^ | Yes | No | Yes | No | Yes | 0 | 0 | 3 |
| **Ravaud et al.,** 1999^45^ | Yes | No | Yes | No | Yes | 0 | 0 | 3 |
| **Young et al.,** 2001^47^ | Yes | No | Yes | Yes | No | 0 | 0 | 3 |
| **Chao et al.,** 2010^49^ | Yes | No | Yes | Yes | Yes | 0 | 0 | 4 |
| **Friedman and Moore,** 1980^50^ | Yes | Yes | Yes | Yes | No | 0 | 0 | 4 |
| **Conaghan et al.,** 2018^44^ | Yes | Yes | Yes | Yes | Yes | 0 | 0 | 5 |
| **McAlindon et al.,** 2017^42^ | Yes | Yes | Yes | Yes | Yes | 0 | 0 | 5 |
| **Mendes et al.,** 2019^41^ | Yes | Yes | Yes | Yes | Yes | 0 | 0 | 5 |
| **Nunes-tamashiro et al**., 2022^43^ | Yes | Yes | Yes | Yes | Yes | 0 | 0 | 5 |
| **Shrestha et al.,** 2018^48^ | Yes | Yes | Yes | Yes | Yes | 0 | 0 | 5 |

*Deduct one point if the method used to generate randomization was described and it was inappropriate.

^Ʈ^ Deduct one point if the study was described as double blind but the method of blinding was inappropriate

**APPENDIX 5.**

**QUALITY OF EVIDENCE – GRADE DETAILED EXPLANATIONS**

1. **Effect on pain compared to placebo**

For all the doses, the quality of evidence is initially considered high as the only accepted studies are randomized controlled trial.

- 1. ***Adjustment for the low dose***

Risk of bias: Downgrade of 1

Since there were only two studies for the low dose and one of them is at higher risk of bias (Gafney and al. with a score JADAD of 1/5), we downgraded the evidence by one.

Inconsistency: No downgrade

There was no significant heterogeneity for the low doses.

Indirectness: No downgrade

All the included studies directly compared intra-articular corticosteroid injection to normal saline, which is a well-accepted placebo.

Imprecision: Downgrade of 1

Since there were only two study of small samples (e.g. <60 participants), we considered there was a significant risk of imprecision.

Publication bias: no downgrade

There were only two studies evaluating lower doses. Therefore, it is difficult to verify the risk of publication bias with a funnel plot. We based the decision to not downgrade the evidence, as when all studies were pooled together in the funnel plot, there was no significant dispersion.

***1.2 Adjustment for the usual dose***

Risk of bias: No downgrade

All of the studies included in this time frame were of high quality, except for Yavuz and al., which was excluded from the analysis. Therefore, we considered there was no significant risk of bias.

Inconsistency: Downgrade of 1

There was a very high heterogeneity for this time frame (I^2^ between 78.9-99%). However, there were multiple explanations for this heterogeneity (see discussion). Therefore, we decided to downgrade the evidence only one point.

Indirectness: No downgrade

All the included studies directly compared intra-articular corticosteroid injection to normal saline, which is a well-accepted placebo.

Imprecision: No downgrade

There was a good number of patients for this time frame (n=785) and multiple studies. The confidence intervals were not too wide, except for the very short term (1-3 weeks), which we did not consider significant enough to downgrade the evidence.

Publication bias: no downgrade

Multiple studies were included in the usual dose subgroup, and they were all well distributed around the funnel plot. Therefore, there was no downgrade.

***1.3 Adjustment for the high dose***

Risk of bias: Downgrade of 1

All three included studies had a 3/5 JADAD score. All studies are on the lower limit to be considered good quality and because the study by Lindsley^1^ is a very small unpublished study, we downgraded the evidence.

Inconsistency: Downgrade of 1

There was only one study in the very short and middle terms. However, in the short term, the three studies showed important inconsistency (I2 = 95.1%). Moreover, the study by Young et a.^2^ had no overlap of his confidence interval with the two other studies. Evidence was downgraded by one.

Indirectness: No downgrade

All the included studies directly compared intra-articular corticosteroid injection to normal saline, which is a well-accepted placebo.

Imprecision: Downgrade of 1

Since there was only one group for very short, short, and middle terms, the three included studies had a small population size (between 8 and 53 participants included). Evidence was downgraded by 1.

Publication bias: no downgrade

There were only two studies evaluating lower doses. Therefore, it is difficult to verify the risk of publication bias with a funnel plot. We based the decision to not downgrade the evidence, as when all studies were pooled together in the funnel plot, there was no significant dispersion.

**Influence of the dose on its effect**

Risk of bias = no downgrade

There was no significant risk of bias to suggest a downgrade.

Inconsistency: Downgrade of 1 for the short term and middle term.

For the very short term, there was no significant heterogeneity.

For the short and middle terms, there was significant heterogeneity (Short term= I^2^ >90% et sign 🡪 -1; Middle term = I^2^ 88% et sign. 🡪 -1).

Indirectness: Downgrade by two for all time frame

All corticosteroid types were converted using conversion table, which causes indirect comparison of corticosteroid type.

Imprecision: downgrade of 1 for the very short term and middle term.

For the very short term, there is only one high dose group and two low dose group, including one of high risk of bias, justifying a downgrade of 1.

For the short term, there were multiple studies for all doses. Therefore, there was no downgrade.

For the middle term, there was only a usual and high dose group, and the high dose group consisted of only one study. Therefore, we downgraded by one.

Publication bias: no downgrade

The results were well distributed in the funnel plot

Sample sizes were judged to be adequate for osteoarthritis population (median of 76 participants based on 116 studies)^4^

Upgrade: no upgrade

There was no large or very large effect size identified. There was no cofounding factor that could have biased the results toward seeing no difference between the doses.

**References**

1. Lindsley H. Treatment of knee osteoarthritis with intra-articular infliximab: ClinicalTrials.gov; 2000 Feb 29 [updated 2018 Febuary 8; cited 2021 August 8th]. Identifier NCT01144143:[Available from: <https://clinicaltrials.gov/ct2/show/results/NCT01144143?view=results1>.

2. Young L, Katrib A, Cuello C, Vollmer-Conna U, Bertouch JV, Roberts-Thomson PJ, et al. Effects of intraarticular glucocorticoids on macrophage infiltration and mediators of joint damage in osteoarthritis synovial membranes: Findings in a double-blind, placebo-controlled study. Arthritis and Rheumatism. 2001;44(2):343-50.

3. Yavuz U, Sökücü S, Albayrak A, Öztürk K. Efficacy comparisons of the intraarticular steroidal agents in the patients with knee osteoarthritis. Rheumatology International. 2012;32(11):3391-6.

4. Copsey B, Thompson JY, Vadher K, Ali U, Dutton SJ, Fitzpatrick R, et al. Sample size calculations are poorly conducted and reported in many randomized trials of hip and knee osteoarthritis: results of a systematic review. Journal of Clinical Epidemiology. 2018;104:52-61.

**Appendix 6. ¸**

**Funnel plots of the effect of intra-articular corticosteroid injection in the very short time, short time, and middle time.**

| A. Very short term  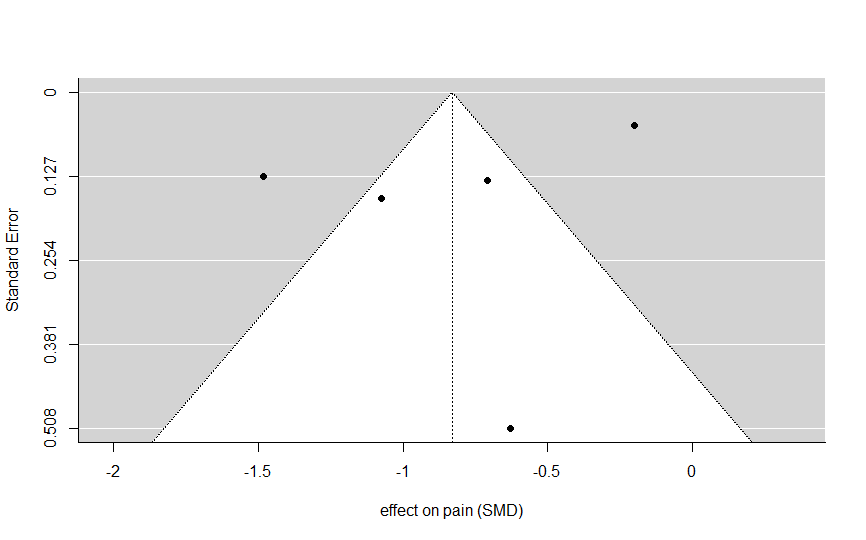 |
| --- |
| B. Short term  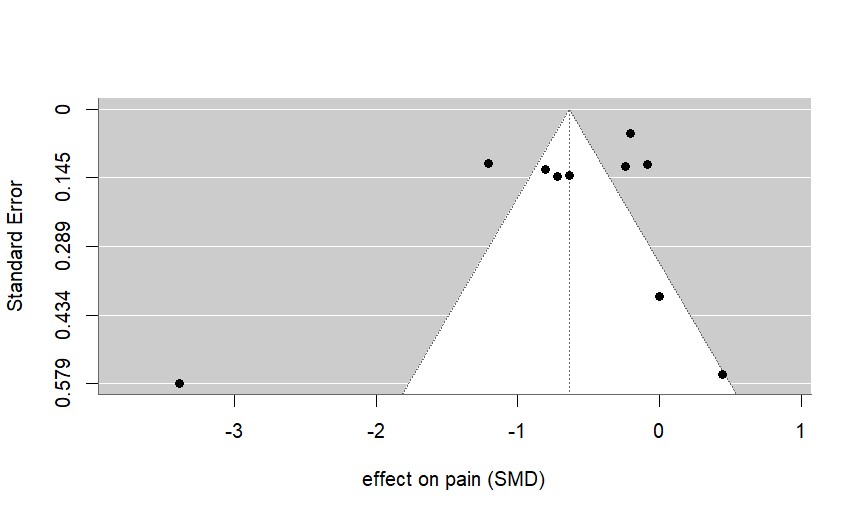 |
| C. Middle term  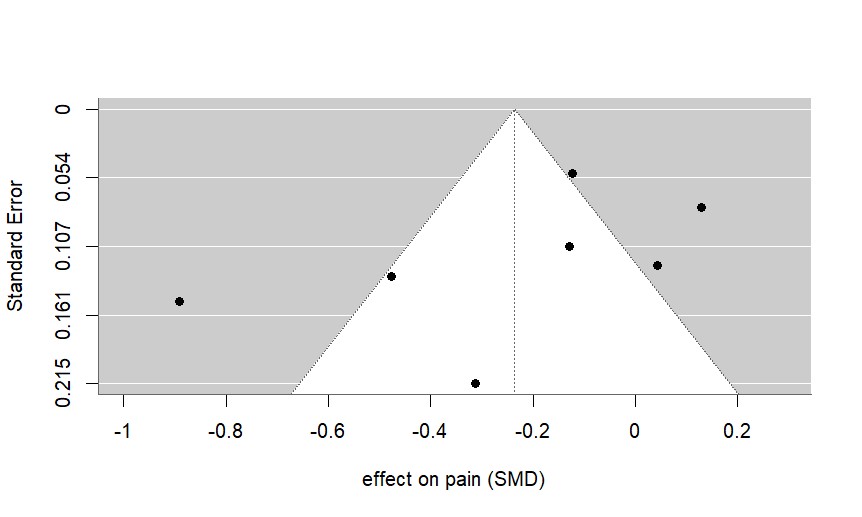 |
